# The role of team dynamics and team acquaintance in group metacognition during medical students’ collaborative learning

**DOI:** 10.1186/s12909-025-07522-y

**Published:** 2025-07-01

**Authors:** Chia-Ter Chao, Yen-Lin Chiu, Chiao-Ling Tsai, Mong-Wei Lin, Chih-Wei Yang, Chiao-Chi Ho, Chiun Hsu, Huey-Ling Chen

**Affiliations:** 1 Division of Nephrology, Department of Internal Medicine, National Taiwan University Hospital, Taipei, Taiwan; 2 Division of Nephrology, Department of Internal Medicine, National Taiwan University College of Medicine, Taipei, Taiwan; 3 Graduate Institute of Toxicology, National Taiwan University College of Medicine, Taipei, Taiwan; 4 Division of Nephrology, Department of Internal Medicine, Min Sheng General Hospital, Taoyuan City, Taiwan; 5 Graduate Institute of Medical Education and Bioethics, National Taiwan University College of Medicine, Taipei, Taiwan; 6 Division of Radiation Oncology, Department of Oncology, National Taiwan University Hospital, Taipei, Taiwan; 7 Department of Surgery, National Taiwan University Hospital, Taipei, Taiwan; 8 Department of Emergency Medicine, National Taiwan University Hospital, Taipei, Taiwan; 9 Division of Chest Medicine, Department of Internal Medicine, National Taiwan University Hospital, Taipei, Taiwan; 10 Department of Medical Oncology, Department of Medical Education & Research, National Taiwan University Cancer Center, Taipei, Taiwan; 11 Department of Pediatrics, National Taiwan University Hospital, No. 1, Sec 1, Ren-Ai Road, Taipei, 100 Taiwan

**Keywords:** Collaborative learning, Medical education, Metacognition, Small group tutorial, Team dynamics

## Abstract

**Background:**

Group metacognition, the capacity to reflect on a group’s cognitive processes, including awareness of other members’ information organization, planning, and efforts for improvement, plays a critical role in collaborative learning efficacy. In medical education, group metacognition supports students in discussing clinical cases, recognizing peers’ diagnostic reasoning, and jointly evaluating their team’s approach to patients care. However, factors influencing group metacognitive competency remain underexplored among medical students. We hypothesized that in collaborative learning, team dynamics, team acquaintance, and instructor support may influence group metacognition.

**Methods:**

In 2021, we recruited medical students from National Taiwan University College of Medicine who participated in a collaborative learning curriculum. We measured the influence of team acquaintance, team dynamics, and instructor support on group metacognition using the Team Collaboration Survey (TCS) and the Group Metacognitive Scale (GMS). Data were analyzed using partial least squares-structural equation modeling (PLS-SEM).

**Results:**

A total of 454 medical students (2nd year, 33.0%; 3rd year, 31.5%; 4th year, 35.5%) were recruited, and 432 (95.2%) completed the survey. PLS-SEM validated three TCS dimensions (team acquaintance, team dynamics, and instructor support) and four GMS dimensions (knowledge of cognition, planning, evaluating, and monitoring). Path analyses revealed significant correlations between both team acquaintance and team dynamics with all four group metacognitive dimensions. However, instructor support showed no significant correlation with metacognitive knowledge and skills.

**Conclusions:**

Strong team dynamics and acquaintance may enhance both metacognitive knowledge and regulation in medical students. Instructors should focus on cultivating interactive group environments that promote effective collaboration. Strategies to strengthen team familiarity and interaction may enhance group metacognition in medical education.

## Introduction

### Individual versus group metacognition: their importance in medical education

Metacognition refers to the process of actively monitoring and regulating one’s cognitive activities [1]. It entails an awareness of and ability to control one’s thought processes, including knowing when and how to apply cognitive strategies for problem-solving. In cognitive psychology, metacognition is often characterized as an executive function that enables learners to retrieve and apply prior knowledge in new contexts [2].

Group metacognition, by contrast, denotes individuals’ ability to reflect on collective cognitive processes within a group, such as recognizing how team members organize information, plan tasks, and evaluate progresses [3]. It captures the essence of group-regulated behavior by focusing on how individuals perceive their influences on group productivity, rather than solely on personal contributions. Group metacognition fosters socially regulated motivation and enhances learners’ engagement during collaborative learning [4].

Emerging from the framework of socially shared metacognition, group metacognition regards metacognitive activity as not only individual but also socially distributed, incorporating group-level monitoring, regulation, and evaluation [5]. Unlike individual metacognition, group metacognition emphasizes the co-construction of shared understanding, joint planning, and mutual regulation, especially crucial for successful collaborative learning.

In medical education, both individual and group metacognition competencies are essential to support proactive learning and improve academic performance [6]. Medical students must adapt to complex clinical environments, meet increasing system and patient demands, and manage uncertainty [7]. Skills such as setting personal learning goals, self-assessment, and developing problem-solving strategies enhance motivation, goal-setting, and decision making quality [8]. In collaborative contexts, group metacognition better prepares medical students for inter-professional teamwork and clinical practice [9]. Prior research suggests that meta-level discourse about group processes correlates more strongly with group outcomes than individual metacognitive skills alone [10]. It can also enhance learning efficacy without increasing group members’ cognitive load [11].

In medical education, while individual metacognition has been extensively studied in relation to clinical reasoning and reflective practice, research focusing on specifically on group metacognition remains limited. This gap is noteworthy, given the emphasis on teamwork, interprofessional collaboration, and shared decision-making in modern healthcare. Investigating group metacognitive process in this context therefore may contribute to a nuanced understanding of how collaborative learning can be optimized in medical education.

In the context of medical education, it is crucial to distinguish between individual metacognition, socially shared metacognition, and group metacognition. Individual metacognition pertains to the learners’ self-awareness and control over personal cognitive strategies. Socially shared metacognition involves co-construction of knowledge through reciprocal regulation, such as when peers prompt each other to re-evaluate diagnosis or challenge clinical assumptions. In contrast, group metacognition focuses on how individual members reflect on the collective cognitive activities of the team, such as jointly assessing whether the team has considered all relevant aspects of a clinical case. In medical problem-solving, group metacognition supports the emergence of shared mental models, distributed reasoning, and collective accountability, all of which are vital for effective team-based care [12].

### Collaborative learning: the role of group factors and their relationship with metacognition

Collaborative learning refers to an educational approach where learners work in groups to solve problems and complete tasks. Each student is responsible for their own learning while gaining from interactions and feedback shared among peers [13]. Based on Social Interdependence Theory [14], this approach posits that the way goals are structured influences member interactions, which in turn shape group outcomes. Positive interdependence, where individuals believe their success depends on others’ success, promotes cooperation and shared responsibility, both of which are central to effective team dynamics and group metacognition. Similarly, Cognitive Elaboration Theory suggests that learning is deepened when individuals explain concepts to one another, elaborate on their thinking, and engage in shared regulation of learning, all of which benefit from strong team acquaintance and high-quality interactions.

In line with Social Interdependence Theory, foundations of team dynamics such as mutual trust, accountability, and cohesion reflect positive interdependence, where group members perceive their success as interlinked. When team members engage in open communication and actively coordinate efforts, they internalize a shared responsibility for the group’s cognitive performance, reinforcing group-level metacognitive behaviors such as co-monitoring and joint evaluation of task strategies. Similarly, Cognitive Elaboration Theory underscores the role of peer explanation, questioning, and argumentation in facilitating deeper learning. Team acquaintance plays a key role in this process by fostering psychological safety and interpersonal comfort, which encourage members to externalize and elaborate their reasoning processes. Familiarity among peers can reduce social inhibitions and increase willingness to offer or solicit clarifications., thereby enhancing opportunities for shared metacognitive regulation. Together, these theories provide a foundation for understanding how interpersonal familiarity and group dynamics do not merely facilitate task completion but actively shape the development of group metacognition.

Despite its promise, collaborative learning can falter if group members struggle with effective teamwork, often due to poor self-regulation or difficulties adapting to collaboration demands, factors frequently linked to emotional or interpersonal issues [15]. Merely participating in collaborative activities does not guarantee development of new knowledge or metacognitive skills. However, peer-to-peer exchanges can initiate shared learning regulation [16]. Group-level factors such as familiarity among members and the quality of interactions are key contributors to collaborative learning success [10]. Social interactions enhance involvement, participation, performance, and learning satisfaction [17]. In medical decision-making, team dynamics are particularly important, as they promote share metacognitive processes that help establish shared mental models, including task goals and relevant knowledge [18]. Such dynamics contribute to group satisfaction and strategic planning, support problem solving, and facilitate evaluation of group processes [19]. Familiarity among members builds a sense of belonging, increasing members’ willingness to reflect on and audit team processes [20], thereby enhancing group performance. Together, these findings underscore the importance of team dynamics and interpersonal relationships in fostering metacognitive growth.

The relevance of group processes in collaborative learning is further amplified by the integration of technologies such as virtual reality (VR) and simulation in medical education. VR can create immersive, engaging environments that demand strong team dynamics and introduce new forms of group metacognitive regulation [21]. Comparative studies between VR-based and traditional learning underscore the importance of understanding how group factors function in technologically mediated contexts [22]. Collaborative VR may enhance knowledge retention and teamwork, making it crucial to understand how group metacognition operates in these settings [23].

Conversely, mismatches in understanding or interpersonal tension can hinder collaboration, while positive emotional dynamics promote trust and team performance [18]. These findings point to the need for intentional efforts to maintain relational balance and shared understanding. The sociability of learning communities, referring to non-task social interactions such as friendship and social support, also plays a role in collaborative learning effectiveness [24]. Poor quality interactions can compromise group learning, leading to reduced productivity, lower interdependence, and weaker metacognitive competence [25]. Social metacognition, the ability to monitor and influence each other’s cognitive strategies, also hinges on the quality of collaboration [26], further supporting the link between group dynamics and metacognitive development.

Despite the importance of team dynamics, interpersonal familiarity, and interaction quality, their specifical influence on the development of group metacognition remains unclear. Most existing studies focus on individual metacognition or overall team performance, with limited attention to how group-level processes shape metacognitive regulation. Few have examined this issue in medical education, where collaborative competencies are critical. This gap underscores the need to examine how team dynamics and acquaintance influence group metacognition among medical students, an aim central to the present study.

### The role of the instructor during collaborative learning and its relationship with metacognition

Factors influencing individual medical students’ metacognitive competency are often context-dependent. In general, learners benefit from explicit instruction and guided practice to become aware of and improve their metacognitive processes. Prior studies have shown that mentoring by experienced tutors can enhance metacognitive skills [27]. Instructors may model clinical reasoning and decision-making, a pedagogical approach akin to “cognitive apprenticeship”, to help students develop metacognitive awareness [28]. Encouraging students to articulate their thinking and providing timely feedback can further foster these skills.

However, learners may not always recognize their own metacognitive processes, a phenomenon referred to as low “metacognitive awareness” [29]. Additionally, factors such as prior experience, task complexity, familiarity, and emotional state can all impact metacognitive functioning [30]. These conditions underscore the value of instructor support in helping students monitor, reflect on, and improve their metacognitive practices.

### Study hypothesis and theoretical framework

Despite the potential significance of group factors, such as team dynamics, member acquaintance, and instructor support in shaping metacognition during collaborative learning, few studies have examined how these elements are associated specifically with group metacognition, defined as the capacity of individual members to reflect on the collective cognitive strategies and processes of the group [3].

**Fig. 1:**
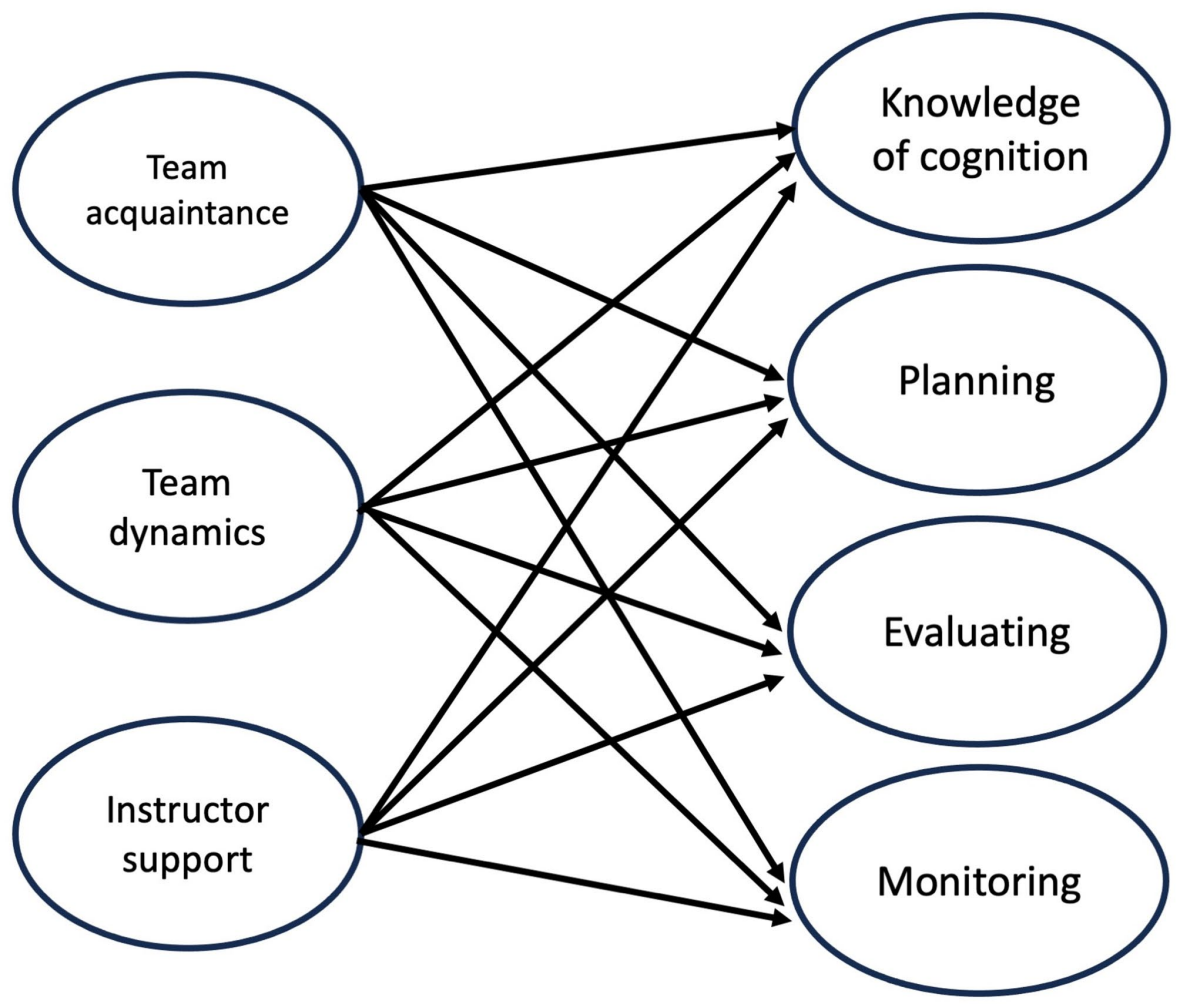
The conceptual framework of this study, which examined the associations between group factors (team acquaintance, team dynamics, and instructor support) and group metacognition (with its four factors, knowledge of cognition, planning, evaluating, and monitoring), among medical students participating in a small group tutorial curriculum

We hypothesized that, in the context of collaborative learning, group-level factors including team acquaintance, team dynamics, and instructor support could significantly influence group metacognitive processes (Fig. 1). A deeper understanding of how these factors interact and affect group metacognition can provide valuable insight to improve the design and efficacy of collaborative learning in medical education.

## Materials and methods

### Ethical statement

The National Taiwan University Hospital’s ethical review board approved the current study’s protocol (NO. 202108011RIND). The board waived the requirement for written informed consent and required verbal informed consent due to the anonymized nature of data collection and the minimal risk to participants. Our study adhered to the principles outlined in the Declaration of Helsinki.

### The context of the current study

We recruited second-, third-, and fourth-year preclinical medical students from National Taiwan University College of Medicine (NTU-CM) during the fall semester of 2021. These students participated in a small group tutorial (SGT) curriculum designed for Years 2 to 4 in our six-year undergraduate medical education program. Unlike other preclinical curricula, which are primarily didactic, the SGT curriculum fosters integrative learning through collaborative discussions involving basic science, clinical medicine, and medical humanities [12].

Before each semester, faculty or senior medical students develop case scenarios adapted from real-world clinical cases for use in SGT sessions. These cases are modified to ensure coherent case flow and to stimulate discussion and reflection. For second-year medical students, the cases focus on medical humanities; third-year cases emphasize physiology and anatomy; and fourth-year cases center on pharmacology and pathology.

At the semester beginning, medical students are randomly assigned into groups of 6 to 10 members using simple random assignment based on number, and paired with an instructor, typically an academic physician affiliated with our medical school. Instructor assignment is independent of subject-matter expertise and is conducted by administrative staff unaffiliated with the study. Randomization also ensures balanced gender distribution across groups.

Weekly group discussions are supported with standardized case materials and guided questions provided in both print and digital formats. Prior to the official SGT sessions, students may opt to meet in person or online to organize their approach and presentation. All instructors have been trained in a pre-curricular workshop, focusing on multiple dimensions relevant to SGT, including teaching skill refinement, instructor-student interactions, experiences sharing from senior instructors, and others. The training emphasized a student-centered, facilitative teaching style, encouraging instructors to adopt techniques such as open-ended questioning, fostering peer-to-peer dialogue, and moderating rather than directing group discussions. These methods were practiced through lectures, interactive group discussion sessions, and peer sharing among instructors. This approach aimed to promote learner autonomy and stimulate reflective thinking rather than content delivery.

During the 2-hour mid-weekly discussion, the instructors participate along with the group of medical students and facilitate case discussions regarding issues including why the case have such presentations, how the diagnoses are made and confirmed, what treatments can be administered, what would the prognosis be like, etc. The instructors act as one of the group discussants and adopt the principle of “tutors as learners”, with flexibility based on group needs. Post-discussion debriefings or feedback are optional, based on each group’s preference.

### Study procedures and study instruments

After participating in at least three SGT sessions, 2nd to 4th year preclinical medical students were invited to complete two validated questionnaires: the Team Collaboration Survey (TCS) [31] and the Group Metacognitive Scale (GMS) [3]. The TCS captures perceptions of teamwork during collaborative learning, while the GMS assesses group-level metacognitive processes. Both instruments were administered anonymously through NTU-CM’s REDCAP online platform.

Students were instructed to complete the surveys simultaneously, either at the end of the third SGT session or within the following two weeks. They were allotted a minimum of 10 min during scheduled time to respond. No significant issues were encountered during the questionnaire completion.

The TCS is a 20-item instrument rated on a 5-point Likert scale (1 = strongly disagree to 5 = strongly agree). It assesses students’ collaborative learning experience across three dimensions [31]:

- **Team acquaintance**, which measures familiarity among members and its effect on teamwork communication and collaboration [32]. An item from this dimension included “My team members share culture-related information to know each other better.”;
- **Team dynamics**, which captures participation, communication, collaboration, trust, and cohesion [33]. An item from this dimension included “Communicating with team members regularly helps me to understand the team project better.”;
- **Instructor support**, which evaluates the perceived guidance provided by instructors during collaborative learning. An item from this dimension included “My team receives guidance during the group discussion session from the instructor.”

These dimensions have been linked to teamwork satisfaction in a prior study [19].

The GMS is a 20-item instrument that measures group metacognition during collaborative learning. It comprises four dimensions [3]:

- **Knowledge of cognition**. An item from this dimension included “We know how to connect new information with prior knowledge.”;
- **Planning**. An item from this dimension included “We organize our time depending on the task.”;
- **Evaluating**. An item from this dimension included “We make judgments on our learning outcomes.”;
- **Monitoring**. An item from this dimension included “We detect and correct errors during group processes.”

Both instruments have demonstrated strong psychometric properties and are appropriate for use in collaborative educational settings.

### Statistical analysis

We conducted all statistical analyses using SmartPLS3 software. Given that factors associated with better team dynamic and metacognition are often context-dependent [34], we began by evaluating the validity and reliability of the TCS and GMS within our educational context. We then used partial least squares-structural equation modeling (PLS-SEM) to examine the influence of medical students’ collaborative learning experiences on their group metacognitive levels.

PLS-SEM was selected due to several methodological advantages. First, it is suitable for prediction-oriented research and allows analysis of complex models with multiple latent constructs, indicator variables, and path relationships, which match the complexity of our proposed model. Second, it is well suited for exploratory studies aiming to expand theoretical frameworks in medical education. Third, it accommodates moderate sample size and non-normal data distributions, offering robust estimation without strict assumptions of normal data distribution [35].

To assess construct validity, we examined both questionnaires’ convergent and discriminant validity. Convergent validity was assessed by factor loadings, average variance extracted (AVE), and composite reliability (CR). Discriminant validity was evaluated using the Fornell-Larcker criterion. We ensured internal consistency reliability with Cronbach’s α. Items with inadequate factor loadings were removed to enhance model fitness.

After validating the measurement model, we performed SEM and path analysis to evaluate the influences of the three TCS dimensions on the four GMS dimensions. Model performance was gauged using R squares, and model fitness was determined by standardized root mean square residual (SRMR) and normed fit index (NFI). Statistical significance was identified if *p* values were below 0.05.

## Results

A total of 454 preclinical medical students participated in the SGT curriculum, comprising 33.0% from the 2nd year, 31.5% from the 3rd year, and 35.5% from the 4th year. Of these, 432 students completed the surveys, yielding a high response rate of 95.2%.

### Validity and reliability of the instruments

PLS analysis revealed that for TCS, the number of items loaded onto the team acquaintance, team dynamics, and instructor support dimensions were three, eight, and three, respectively (Table 1). All 14 identified items had loadings higher than 0.8. The CR and AVE for the three dimensions exceeded 0.6, supporting validity of TCS. These dimensions, including team dynamics, team acquaintance, and instructor support, demonstrated good internal consistency, with Cronbach’s α values above 0.8 (Table 1). Regarding GMS, the knowledge of cognition, planning, evaluating, and monitoring dimensions had five, five, four, and four items loaded onto them, respectively. All 18 items had loadings greater than 0.7. The CR and AVE for the four GMS dimensions were above 0.9 and 0.6, respectively. These dimensions also showed good internal consistency, with Cronbach’s α values ranging from 0.881 to 0.913 (Table 1). We also assessed the discriminant validity of the TCS and GMS dimensions using the Fornell-Larcker criterion, yielding favorable measurement outcomes (Table 2).

**Table 1:** Validity and reliability analyses of team collaboration survey and group metacognition scale

Dimensions/items	Loading	Composite reliability	Average variance extracted	Rho value	Cronbach’s α value
**Team Collaboration Survey**					
*Team acquaintance*		0.907	0.765	0.846	0.846
TA1. My team members share personal information in order to know each other better.	0.896				
TA2. My team members share culture-related information to know each other better.	0.887				
TA3. Getting to know one another in my team allows me to interact with teammates more efficiently.	0.841				
*Team dynamics*		0.956	0.73	0.947	0.947
TD1. My team members learn how other team members wish to be treated and then respond accordingly.	0.879				
TD2. My team members reply all responses in a timely manner.	0.878				
TD3. Communicating with team members regularly helps me to understand the team project better.	0.873				
TD4. My team trusts each other and works toward the same goal.	0.856				
TD5. My team members encourage open communication with each other.	0.855				
TD6. My team members clearly know their roles during the collaboration process.	0.838				
TD7. My team is receiving feedback from each other.	0.83				
TD8. My team develops cleat collaborative patterns to increase team learning efficiency.	0.822				
*Instructor Support*		0.925	0.805	0.88	0.879
F1. The instructor acts as a referee when our team members cannot seem to resolve differences.	0.908				
F2. My team is receiving guidance during the group discussion session from the instructor.	0.903				
F3. The support from the instructor helps my team reduce anxiety among team members.	0.881				
**Group metacognition scale**					
*Knowledge of cognition*		0.933	0.735	0.91	0.909
K1. We know how to use the material.	0.888				
K2. We know how to organize the new information.	0.884				
K3. We know how to select relevant information.	0.883				
K4. We know how to connect new information with prior knowledge.	0.83				
K5. We know our strength as learners.	0.799				
*Planning*		0.914	0.679	0.884	0.881
P1. We select the appropriate tools.	0.879				
P2. We identify the strategies depending on the task.	0.866				
P3. We determine what the task requires.	0.811				
P4. We organize our time depending on the task.	0.782				
P5. We plan the activities.	0.778				
*Evaluating*		0.939	0.793	0.914	0.913
E1. We make judgments on the teamwork process.	0.91				
E2. We make judgments on the instruments.	0.903				
E3. We make judgments on our learning outcomes.	0.901				
E4. We make judgments on the workload.	0.845				
*Monitoring*		0.926	0.757	0.895	0.893
M1. We improve our work with group processes.	0.893				
M2. We check our approach to improve our outcomes.	0.881				
M3. We detect and correct errors during group processes.	0.863				
M4. We ask questions to check our understanding.	0.843				

*GMS*,* group metacognition scale*

**Table 2:** Discriminant validity analysis results

Dimensions	Team acquaintance	Team dynamics	Instructor support	Knowledge of cognition	Planning	Evaluating	Monitoring	Student’s grade
Team acquaintance	**0.875**							
Team dynamics	0.836	**0.854**						
Instructor support	0.71	0.756	**0.897**					
Knowledge of cognition	0.663	0.712	0.602	**0.858**				
Planning	0.663	0.714	0.596	0.756	**0.824**			
Evaluating	0.652	0.719	0.587	0.795	0.779	**0.89**		
Monitoring	0.648	0.702	0.573	0.785	0.789	0.816	**0.87**	
Student’s grade	-0.021	0.029	0.158	0.034	0.057	-0.037	-0.016	**1**

### Factors correlated with group metacognition

We evaluated the relationship between the dimensions of students’ collaborative learning process and their group metacognition using path analysis, taking into account student’s grade. There was no collinearity between the dimensions of TCS and GMS, as indicated by low variance inflation factors (ranging from 1.678 to 3.728). The SRMR, NFI, and RMS theta values for our SEM were 0.041, 0.865, and 0.123, respectively. According to established criteria for SRMR (< 0.08) [36] and NFI (> 0.8) [37], our model demonstrated good fit.

**Fig. 2:**
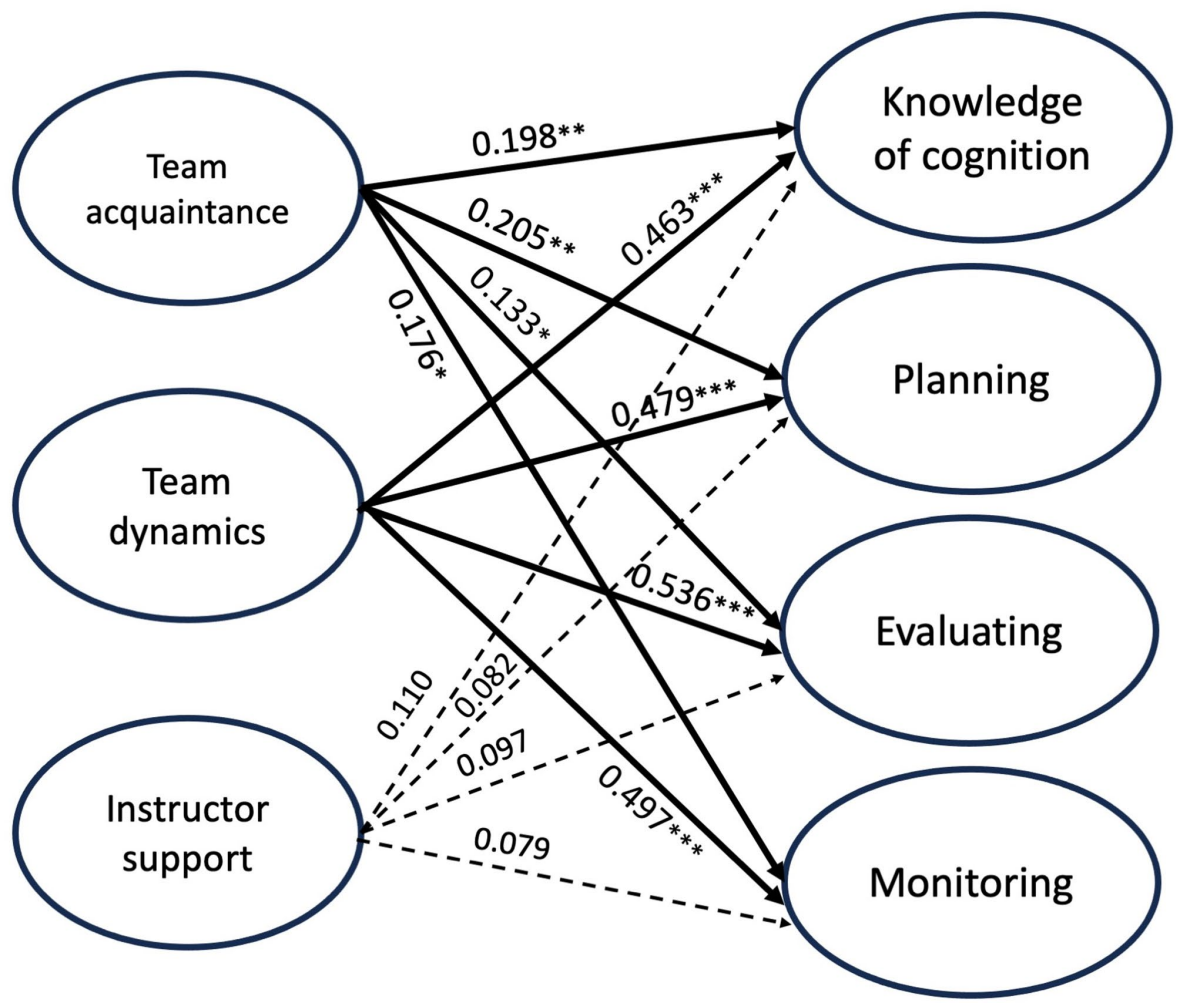
The partial least squares-structural equation modeling results for the measurement and the structural model. Numbers near the arrow indicate path coefficients. *p < 0.05; **p < 0.01; ***p < 0.001

Table 3 displays the path coefficients and relationships between collaborative learning and metacognitive dimensions, while Fig. 2 presents the path analysis. We discovered that team acquaintance was significantly correlated with several metacognitive dimensions among medical students, including knowledge of cognition (*p* = 0.002), planning (*p* = 0.005), evaluating (*p* = 0.042), and monitoring (*p* = 0.012). Similarly, team dynamics showed a significant correlation with knowledge of cognition (*p* < 0.001), planning (*p* < 0.001), evaluating (*p* < 0.001), and monitoring (*p* < 0.001). However, instructor support did not show a correlation with any metacognitive dimension, as indicated in Table 3. Similarly, student’s grade did not exhibit correlations with any metacognitive dimension.

**Table 3:** Results from path analysis

	Path coefficients	*t*	*P* value
*Domain: Knowledge of cognition (* *R* *square = 0.527; Adjusted* *R* *square = 0.522)*			
Team acquaintance	0.198	3.169	*0.002*
Team dynamics	0.463	6.969	*< 0.001*
Instructor support	0.110	1.855	*0.064*
Student’s grade	0.007	0.203	*0.839*
*Domain: Planning (R square = 0.529; Adjusted R square = 0.525)*			
Team acquaintance	0.205	2.845	*0.005*
Team dynamics	0.479	6.057	*< 0.001*
Instructor support	0.082	1.080	*0.281*
Student’s grade	0.034	1.084	*0.279*
*Domain: Evaluating (R square = 0.532; Adjusted R square = 0.527)*			
Team acquaintance	0.133	2.037	*0.042*
Team dynamics	0.536	7.927	*< 0.001*
Instructor support	0.097	1.608	*0.235*
Student’s grade	-0.065	1.608	*0.051*
*Domain: Monitoring (R square = 0.508; Adjusted R square = 0.503)*			
Team acquaintance	0.176	2.535	*0.012*
Team dynamics	0.497	6.710	*< 0.001*
Instructor support	0.079	1.190	*0.235*
Student’s grade	-0.039	1.207	*0.228*

## Discussion

In this study, we surveyed 432 preclinical medical students (Years 2–4) following participation in a collaborative SGT curriculum and examined how elements of the group learning process related to group metacognitive competencies. Our findings revealed that both team acquaintance and team dynamics were significantly associated with all dimensions of group metacognition, while instructor support was not. These results suggest that student perceptions of peer relationships and team functioning play a critical role in supporting group metacognitive development. In contrast, instructor involvement, as currently structured, appears less influential in shaping these outcomes.

### Role of team acquaintance

Group-based learning frequently begins with brainstorming and proceeds through collaborative problem-solving. “Group process fluency”, the ease with which members work together [38], is shaped by interpersonal familiarity, shared confidence, and mutual understanding. Conversely, poor relationships and interpersonal tension can hinder communication, reduce group effectiveness, and lead to maladaptive metacognitive responses, such as suboptimal work strategies (e.g. overconfidence or disengagement) [39]. Enhancing team acquaintance may thus improve group process fluency and raise collective awareness, both of which support metacognitive functions.

Familiarity within the group promotes psychological safety, increases trust, and enables members to more openly share feedback and reflect on team processes. These conditions foster a richer collaborative learning environment. The benefits of team familiarity may increase over time, particularly with repeated task engagement and evolving group identity. Strategies to build team familiarity, such as stable group composition, structured icebreakers, or informal social interactions, can enhance group metacognitive development without compromising creativity or critical thinking.

For instance, a comparative study has shown that technology-enhanced interactive groups engaged earlier in shared planning and reflection, leading to stronger metacognitive performance compared to teacher-led groups [18]. Structured opportunities for peer interaction, combined with feedback and reflective discussion, appear to be key strategies for enhancing group metacognitive skills in medical education [40]. These strategies may better equip students for the teamwork demands in healthcare contexts through fostering further collaborative learning and enhancing their group metacognitive skills.

### Role of team dynamics

Team dynamics refers to the evolving, interdependent interactions among group members working toward a shared goal. These dynamics are shaped by time, environment, and group cohesion [41]. Team dynamics promote members’ group metacognition through enhancing “social metacognition,” where metacognition demands are distributed among group members and individuals monitor, evaluate, and support each other’s cognitive strategies, leading to improved efficiency, motivation, and emotional regulation [5].

Team development typically progresses through distinct stages: formation (self-awareness, building familiarity and role awareness), task and role compilation (recognizing individual responsibilities, skills and their contributions to task achievement), team compilation (establishing interdependence and role distribution), and team maintenance (sustaining effective team functioning) [42]. Each stage supports specific aspects of group metacognition, including planning, monitoring, and evaluation. Consequently, team dynamics are an essential factor in enhancing metacognition, particularly in the group collaboration context.

Effective team dynamics are essential for successful collaboration. A recent qualitative study identified three factors (team building, team work, and team performance) are critical contributors to productive group learning and optimized team dynamics [43]. High-functioning teams promote mutual trust, social cohesion, and strategic problem-solving, all of which support reflective learning and metacognitive growth. Upholding team dynamics therefore assist in promoting learner satisfaction and potentially enhance learning efficacy through cultivating collaboration skills.

To improve group metacognition, facilitators may consider implementing structured guidance and group regulation strategies [44], including a mix of feedback and feedforward. Combining feedback and feedforward can assist in co-creating learning goals and enhancing social dynamics. Feedback improves goal-setting and self-regulation, whereas feedforward prepares for future tasks and fosters a positive learning environment, leading to better group regulation, motivation, and metacognition in supported groups [45]. As an example, instructors may use dialogic feed forward, instead of written one, during their interactions with students amidst or after each SGT session [46], creating positive group atmosphere and strengthening students’ learning engagement. Students can be encouraged to co-produce teaching materials beyond the original content [47]. Although these approaches may entail more labor from all members and create emotional challenges for instructors [46], they can provide under-recognized benefits with regard to group metacognition in the long run.

Some actionable strategies, such as structured rotating roles, team-building activities, icebreaker exercises, and flipped-classroom designs, can be readily implemented to support team functioning and group reflection. Instructors may consider using these tools to enhance both group cohesion and the regulation of group metacognition.

### Role of instructor support

The absence of a significant relationship between instructor support and group metacognition in our study is noteworthy. One possible explanation is that students may perceive their peers as the primary source of support and learning, functioning as “peer tutors” in a reciprocal teaching model [48]. Prior research has shown that students often organize themselves into a peer-assisted learning structures, especially when working within the same academic year, where learners naturally alternate between teaching and learning roles [49].

In our curriculum, the presence of pre-designed cases and structured discussion prompts may have provided sufficient scaffolding for students to engage effectively without relying heavily on instructor facilitation. Instructors’ roles may be overshadowed by peer tutoring, diminishing students’ perception of active facilitation. Moreover, students may expect more support from the instructors in collaborative learning style curricula, whereas instructors, on the contrary, tend to emphasize self-directed learning in such curricula. This mismatch between student expectations and instructor intentions, in combination with the peer learning structure of our education context and the availability of education materials, may help explain why instructor support was not associated with higher group metacognitive scores.

Nonetheless, this finding contrast with previous research in non-medical educational settings, where instructor facilitation has been shown to support metacognitive development by modeling thinking process and prompting reflection [50]. It is possible that the hierarchical educational culture prevalent in East Asia, where instructors are traditionally seen as authoritative figures, may influence student-instructor dynamics in ways that diminish perceived instructor contributions to collaborative learning. Alternatively, the unique dynamics of medical students’ peer interactions or the structure of our curriculum may be responsible as well. The specific instructional model adopted, namely, the “tutor as learner” approach in our system, in which instructors act as co-participants rather than explicit facilitators, plays a role as well. While this model encourages egalitarian dialogue, it may result in missed opportunities for instructors to intentionally prompt metacognitive reflection or to structure activities that promote monitoring and evaluation of group thinking. In contrast, more directive roles, such as explicitly modeling metacognitive questioning or guiding post-discussion reflections, can help elevate the instructor’s role in enhancing group metacognition.

In light of these nuances, instructors may need to shift from content facilitators to environment designers, using technology and structured scaffolds to foster rich, interactive discussions [24, 51]. A metacognitive environment (e.g., construct problem-solving activities including problem structuring, scaffolding, scripting, as well as discourse supports) can encourage students to engage more deeply in collective reflection and self-regulation [52]. Thus, the role of the instructor is not to solely participate in the interactive discussion but also attempt to construct an environment conducive to effective interactions. By doing so, instructors can enhance collaborative learning outcomes, ensuring that the group’s efforts lead to better group metacognitive competency.

### Pedagogical implications

Our findings underscore the need for pedagogical strategies that directly reflect the factors most strongly associated with group metacognition, namely, team acquaintance and team dynamics. The observed positive associations suggest that fostering stable, familiar group environments can enhance learners’ comfort with engaging in collective reflection and regulation. Therefore, maintaining consistent group membership across multiple sessions may allow trust and shared mental models to develop more fully. The significant role of team dynamics suggests the importance of cultivating high-quality interpersonal and task-related interactions. Educators may consider implementing peer-led facilitation, structured role rotation, and shared goal-setting to promote active engagement and reciprocal accountability. These approaches align with our data indicating that teams with stronger internal processes are more likely to demonstrate higher metacognitive awareness and regulation.

Instructors can consider redesigning their roles, from direct facilitating the SGT curriculum, to facilitating the metacognitive environments that support peer regulation, dialogue, and reflection on the group process. Incorporating dialogic feedforward, promoting the co-production of learning materials, and rotating team roles may further enhance group metacognitive levels. Instructors may also consider strategies such as structured icebreaker activities to foster team acquaintance or role rotations to improve team member interactions. In addition, a creative interactive discussion environment can entail techniques such as facilitators assigning medical students to co-lead case discussions or using side-by-side digital platforms for real-time collaborative problem-solving. These pedagogical approaches may better prepare these medical students for teamwork-centric healthcare settings.

Technology-enhanced collaborative learning, an educational context increasingly encountered over time, may also be influenced by alterations in group factors and students can have varied levels of group metacognition. E-learning or online environment creates distinct atmosphere that positively or negatively affects participating students’ engagement, depending on learning characteristics [53]. Our findings regarding fundamental group processes may be applicable to online, hybrid, VR-based, or other immersive technology-based collaborative settings [21–23]. Given the expanding use of novel teaching and learning scenarios that promote group learning such as interprofessional educational activities and workplace-based competency training, we expect that our findings can be further explored or adapted for these contexts.

### Strengths, limitations

This study has several strengths. We enrolled a large and representative sample, covering over 90% of the preclinical student cohort at a major medical school, providing strong statistical power for instrument validation and structural modeling. The use of rigorous statistical methods, including PLS-SEM, enabled us to uncover nuanced relationships between group learning factors and group metacognitive competencies. Our study is one of the first to empirically demonstrate that both team acquaintance and team dynamics are significantly associated with all dimensions of group metacognition among medical students in a collaborative learning context. This finding expands the theory of group metacognition in small group tutorial contexts.

However, some limitations should be acknowledged. First, the study was conducted at a single institution, which may limit the generalizability of the findings to other settings with different curricula or educational cultures. Second, the cross-sectional design precludes causal inference and limits our ability to assess changes in group metacognition over time. Longitudinal studies are needed to evaluate how these relationships evolve. Third, students’ self-reported perceptions may have been influenced by contextual variables such as task complexity, group composition, and environmental or cultural expectations. Although participants were asked to rate their average experiences, such variability could still introduce bias. Fourth, group dynamics, acquaintance, and metacognitive development may function differently in digital or hybrid collaborative learning environments, which were not explored in this study. Institutional and socio-cultural features, and the degree of peer interactions may also influence the association we observed [54]. From a future perspective, we suggest conducting cross-cultural comparison studies on group metacognition in collaborative learning to demonstrate the generalizability of the findings from this study. Lastly, unmeasured confounders may have impacted the study’s findings. Further research is needed to strengthen and enrich our findings. Longitudinal studies to track the changes and modifying factors of group metacognition over time, and the long-term impact of interventions aiming to improve team acquaintances and dynamics are encouraged as well.

Additionally, our findings may reflect underlying cultural influences inherent in East Asian educational contexts, where collectivist values, strong group orientation, peer interdependence are prominent. In such settings, students may naturally rely more on group cohesion and peer interactions during collaborative learning, while the instructor’s role is often perceived as distant or authoritative, rather facilitative. This cultural backdrop may explain the stronger influence of team dynamics and team acquaintance on group metacognition observed in our study. The generalizability of our findings to other cultural background, such as Western society where learner autonomy and instructor-led facilitation are more emphasized, deserves to be evaluated in future studies.

## Conclusion

In this study, we surveyed nearly 500 medical students participating in a SGT curriculum to assess how group processes related to group metacognitive levels. We discovered that team acquaintance and team dynamics were significantly associated with students’ group metacognitive knowledge and regulatory skills, whereas instructor support was not. These finding suggest that fostering familiarity and positive interactions among medical students within groups may enhance collaborative learning by strengthening group metacognition. Instead of leading discussions, instructors should focus on creating a learning environment that encourages team acquaintance and team dynamics, which in turn enhances group metacognition. Future research can explore how these group factors function across different stages of medical training, specialties, or cultural and institutional contexts to better understand the generalizability and mechanisms of group metacognition development.

## Data Availability

The raw data for conducting this analysis are available upon reasonable request to the corresponding author.

## Abbreviations

AVE: Average variance extracted
CR: Composite reliability
GMS: Group metacognitive scale
NFI: Normed fit index
NTU-CM: National Taiwan university college of medicine
PLS-SEM: Partial least squares-structural equation modeling
RMS: Root mean square
SGT: Small group tutorial
SRMR: Standardized root mean square residual
TCS: Team collaboration scale
